# The Gut and Skin Microbiome and Its Association with Aging Clocks

**DOI:** 10.3390/ijms25137471

**Published:** 2024-07-08

**Authors:** Mildred Min, Caitlin Egli, Raja K. Sivamani

**Affiliations:** 1 Integrative Skin Science and Research, 1451 River Park Drive, Suite 222, Sacramento, CA 95819, USAcaitlin@integrativeskinresearch.com (C.E.); 2 College of Medicine, California Northstate University, 9700 W Taron Dr, Elk Grove, CA 95757, USA; 3 College of Medicine, University of St. George’s, University Centre, West Indies, Grenada; 4 Integrative Research Institute, 4825 River Park Drive, Suite 100, Sacramento, CA 95819, USA; 5 Pacific Skin Institute, 1495 River Park Drive, Sacramento, CA 95815, USA; 6 Department of Dermatology, University of California-Davis, 3301 C St #1400, Sacramento, CA 95816, USA

**Keywords:** aging clocks, microbiome, epigenetic, metagenomic, biological age, chronological age

## Abstract

Aging clocks are predictive models of biological age derived from age-related changes, such as epigenetic changes, blood biomarkers, and, more recently, the microbiome. Gut and skin microbiota regulate more than barrier and immune function. Recent studies have shown that human microbiomes may predict aging. In this narrative review, we aim to discuss how the gut and skin microbiomes influence aging clocks as well as clarify the distinction between chronological and biological age. A literature search was performed on PubMed/MEDLINE databases with the following keywords: “skin microbiome” OR “gut microbiome” AND “aging clock” OR “epigenetic”. Gut and skin microbiomes may be utilized to create aging clocks based on taxonomy, biodiversity, and functionality. The top contributing microbiota or metabolic pathways in these aging clocks may influence aging clock predictions and biological age. Furthermore, gut and skin microbiota may directly and indirectly influence aging clocks through the regulation of clock genes and the production of metabolites that serve as substrates or enzymatic regulators. Microbiome-based aging clock models may have therapeutic potential. However, more research is needed to advance our understanding of the role of microbiota in aging clocks.

## 1. Introduction

Aging is a complex biological process with hallmark internal and external characteristics. Intrinsic aging, otherwise known as biological aging, is the result of cellular and molecular changes, genetics, and hormonal changes leading to the progressive loss of physiological integrity and impaired functionality [1]. Extrinsic aging is influenced by external exposure to the sun, pollution, dietary habits, and other lifestyle factors that accelerate normal physiologic aging. The most common measurement of age is chronological age, which is based on the time from birth to a given date. However, the use of chronological age to describe aging does not consider interindividual variations in genetic susceptibilities, environmental exposures, genetics, dietary habits, and other factors that may accelerate physiological aging. An alternative metric that has evolved rapidly is the estimation of biological age utilizing age-associated changes, namely, the use of aging clocks and mortality timers. The discrepancy between the predicted biological age and chronological age, known as the age gap metric, provides additional insight on how the pace of an individual’s biological aging may not match their chronological age [2]. For example, a greater biological age may indicate accelerated aging compared to chronological age. Differentiating chronological age from biological age is important, as it may guide clinical decision-making and risk assessment, such as in cases assessing cancer risk [3].

Aging clock models utilize age-related changes to create a reliable estimation of biological age. Aging clocks are created from biological systems that change with age, including epigenetic changes, microbiome composition, blood biomarkers, telomere length, glycomic levels, and more. Notably, numerous epigenetic aging clocks have been developed to track biological age. For example, the Hunnam clock tracked changes in DNA methylation via cytosine phosphate guanine (CpG) islands, and the Horvath clock was clarified to include more tissues and CpG island patterns [4,5].

**Figure 1:**
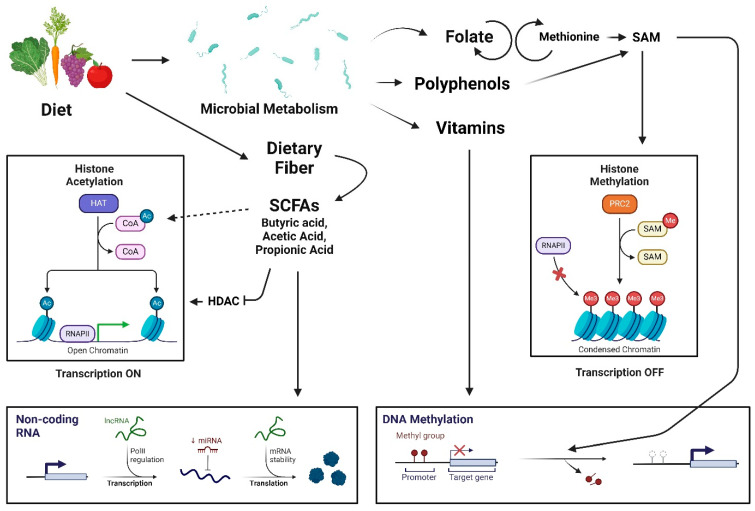
Diet-dependent epigenetic modifications by intestinal microbiota. Microbial metabolism regulates pathways involved with histone methylation, DNA methylation, histone acetylation, and non-coding RNAs. SAM, S-adenosylmethionine; HAT, histone acetyltransferase; Ac, acetyl; CoA, coenzyme A; RNAPII, RNA polymerase II; SCFA, short-chain fatty acid; lncRNA, long non-coding RNA; PolIII, polymerase III; miRNA, micro RNA; mRNA, messenger RNA; PRC2, polycomb repressive complex 2; Me, methyl; HDAC, histone deacetylase. The dotted arrow represents a minor action. This figure was created by BioRender.com.

The role of skin and gut microbiota (the microbiome) extends beyond barrier and immune function. Recent studies have shown that the microbiome may influence aging [6,7]. The microbiome is involved in the metabolism of host proteins and lipids, thereby producing secondary bioactive products that may be involved in regulating aging clocks [8]. Epigenetic modifying enzymes, for instance, require specific substrates to catalyze changes to the chromatin, which the microbiota can provide. For example, the microbial metabolism of dietary fiber has been shown to produce short-chain fatty acids (SCFAs) that are involved in regulating histone acetylation and non-coding RNAs (Figure 1) [9,10]. Furthermore, gut microbiota has been shown to regulate histone modifications in a diet-dependent manner [10]. Thus, the role of microbiota in epigenetic modifications may influence such aging clocks, which predict age based on the detection and quantification of epigenetic markers, such as DNA methylation.

Studies have also shown that microbiome composition, diversity, and functionality shift with age [11], and there have been several microbiome-based aging clocks developed as a result. For example, age-related functional characteristics of the gut microbiome may include decreased vitamin B12 synthesis, reduced reductase activity, increased DNA damage, and reduced immune function [12]. Additionally, there are several differences in gut microbiome composition and phenotype with age, and these changes also reflect the role of the microbiome in aging. For example, aging has been associated with a loss of diversity in core microbiota groups [13], while increased longevity has been associated with increased taxonomic alpha diversity [14]. As a result, several studies have sought to utilize the gut microbiome to create aging clocks, and other studies have demonstrated how the gut microbiome may influence non-microbiome-based aging clocks. Studies have also demonstrated skin microbiome changes with age, and key findings include higher bacterial alpha diversity [15], variations in *Corynebacterium*, *Acinetobacter*, and *Cutibacterium* among younger versus older adults [16], and correlations between the facial skin microbiome and variations in sebum and hydration levels [17]. The utilization of the skin microbiome to estimate biological age is still emerging. Aging clocks based on microbiome taxonomy, functional pathways and enzymes, and the meta metabolome not only predict biological age but also give insight into the mechanisms by which the microbiome affects host aging and aging clock predictions. Recent advancements in whole genome sequencing and deep learning models have also made it more efficient to create these predictions and validate findings.

Despite recent advancements in technology, there remains a gap in knowledge in understanding how the skin and gut microbiota influence aging clocks. This review aims to summarize the most relevant studies demonstrating how these microbiota influence aging clocks as well as clarify the differences between chronological and biological age.

## 2. Methods

A literature search of PubMed/MEDLINE was performed for articles prior to 22 January 2024 with the following relevant keywords using the Advanced Search Builder tool: (“skin microbiome” OR “gut microbiome”) AND (“aging clock” OR “epigenetic”). After a preliminary review of the resulting articles, those with relevant subject matter were retrieved for full-text review. Articles that presented gut and skin microbiome-derived aging clock models and their validation metrics were included. Review articles, case reports, case series, and other studies not adequately describing microbiome aging clocks were excluded. Furthermore, studies that were not written in the English language were excluded. During the full-text review, additional articles were retrieved after scanning the references for relevant reports. Only articles presenting results from human studies were included.

## 3. Results and Discussion

### 3.1. Literature Search

The initial search resulted in 2381 full-text articles. Two independent reviewers assessed the literature and data from each article, and 63 articles were included in the results or discussion based on relevance or as a result of scanning references during the full-text review. Of these 63 articles, 7 were found to describe gut or skin microbiome-derived aging clocks (Table 1). Furthermore, the predictability of these aging clocks against chronological age is described in Table 2.

### 3.2. Gut Microbiome Aging Clocks

#### 3.2.1. Taxonomic Clocks

Taxonomic gut microbiome aging clocks aim to utilize the characterization of the microbiome to associate differences with age. One 2020 study created a human gut microbiome aging clock based on taxonomic profiling utilizing deep learning [18]. In this study, over 4000 metagenomic profiles were aggregated from individuals aged 18 to 90 years, and the Deep Neural Network (DNN) was chosen as the model of choice to detect associations between age and microbiome composition. An aging clock was created based on these parameters with a mean absolute error of 10.6 years. On further analysis of gut microbiome characterization, it was found that most microbes were able to shift the predicted age by less than 0.5 years, and the microbes with the biggest influence were mainly SCFA-producing bacteria, such as *Bifidobacterium* spp., *Akkermansia muciniphila*, and *Bacteroides* spp. or pathogenic bacteria, such as *Escheria coli* and *Campylobacter jejuni* [18]. SCFA-producing bacteria, such as *A. muciniphila*, have been shown to improve gut barrier integrity and modulate metabolism, inflammation, and immune function [24]. Alternatively, *C. jejuni* infection is the leading cause of gastroenteritis and can activate inflammatory pathways in the gut [25]. Interestingly, higher relative abundances of *A. muciniphila* shifted the predicted age to a younger age than the chronological age, while *C. jejuni* shifted the predicted age to higher than the chronological age.

Another 2020 study evaluated different human microbiomes to assess their ability to predict aging [19]. Utilizing the 16S rRNA sequencing data from a total of 4434 fecal samples of healthy subjects aged 18 to 90 years, the study utilized random forests to regress the relative abundance of microbiota against the subjects’ chronological ages. The gut microbiota was found to be correlated with chronological age, with a mean absolute error of 11.5 years. Interestingly, there were several taxa that contributed more to the age prediction model than others. In the gut, bacteria from the genera *Bifidobacterium* and *Blautia* or the families *Lachnospiraceae*, *Ruminococcaceae,* and *Clostridiaceae* were consistently found to have high feature importance scores [19]. *Bifidobacterium* and *Blautia* are SCFA-producers that have been shown to modulate aging-related oxidative stress, improve intestinal barrier function, and modulate neurocognitive abilities via the gut–brain axis, among other notable beneficial effects [26,27,28,29]. Similarly, *Lachnospiraceae* and *Ruminococcaceae* have been shown to be highly occurring, symbiotic bacteria associated with longevity, especially in semi-supercentenarians [30]. Clostridiaceae have also been shown to be less abundant in neurodegenerative disorders, such as Alzheimer’s disease [31]. Many of these bacteria are also counted when calculating the Firmicutes/Bacteroides ratio [32], which is a relevant marker of gut dysbiosis. Future studies may build on these findings and include expanded populations as well as evaluate the rate of aging based on differences between predicted biological age and chronological age. Furthermore, the use of 16S rRNA sequencing may limit the characterization of the microbiome in comparison to whole genome sequencing techniques.

#### 3.2.2. Biodiversity Clocks

Microbiome biodiversity may also indicate biological aging. For example, a 2020 study analyzed 1649 publicly available 16S rRNA sequencing data sets containing a wide range of subjects, including elderly and young subjects (0 to 109 years) with various health statuses and ethnicities [20]. They aimed to create a model that estimates age based on biodiversity indices and utilized the Relative Species Abundance distribution (RSA) modeling approach. This study found a decrease in microbial diversity with unhealthy phenotypes at older ages [20]. Another 2021 study evaluated the gut microbiome and phenotypic data from over 9000 individuals aged 18 to 101 years and found that the β-diversity of gut microbiomes becomes more unique with age and that the increased uniqueness of gut microbiomes in people over 84 years old is related to metabolomic changes that have already been associated with longer lifespan, such as phenylalanine/tyrosine microbial fermentation products [21,33]. These studies show the significance of microbiome biodiversity in the aging process and their potential implications on aging clock predictions; however, more research is warranted to create predictions based on these findings that can be correlated against chronological age.

#### 3.2.3. Functional Clocks

Building on taxonomic and biodiversity clocks are functional clocks, which create aging predictions based on both composition and functionality. These aging clocks require metagenomic sequencing to characterize taxonomy as well as metabolic pathways and enzymes. Moreover, the combined analysis of the two may refine aging predictions. One 2022 study analyzed 4478 fecal samples from individuals greater than 18 years of age to analyze the influence of age and other factors on the taxonomy and functional profiles of the gut microbiota [22]. Based on taxonomic profiles alone, the best age prediction model created had a mean absolute error of 9.5 years. Based on metabolic pathway profiles alone, the best-curated aging model had a mean absolute error of 10.2 years. When integrating the taxonomic and functional models and adjusting for host confounding factors utilizing machine learning, the model created had a mean absolute error of 8.3 years. Individual features of interest were also identified to determine the most influential taxonomic and functional features of aging. Among all microbial species and metabolic pathways, the most predictive factors were acetyl-CoA biosynthesis, nicotinate degradation, and *Finegoldia magna*. Microbial species that increased with age included *F. magna*, *Bifidobacterium dentium*, and *Clostridium clostridioforme*, while *Prevotella copri* and *Burkholderialse* bacterium decreased with age. Similarly, acetyl-CoA biosynthesis, L-leucine degradation, and nicotinate degradation increased with age, while taxadiene biosynthesis, tRNA processing, and L-isoleucine biosynthesis decreased with increasing age [22]. Many of these findings align with previous studies that have detected age-related changes in microbial taxonomy and functionality. For example, *F. magna*, which increased with age, has been associated with arthritis [34]. The reduced relative abundance of *Prevotella* has also been associated with aging and frailty [35]. Furthermore, the consumption of branched-chain amino acids has been found to increase with age, and this correlates with the increase in leucine metabolism and reduction in the isoleucine synthesis pathway found in the study [36].

Another 2024 study evaluated the gut microbiome profiles of 90,303 fecal samples from individuals aged 0 to 104 years with a wide range of health statuses and lifestyle habits [23]. The metagenomic gut microbiome aging clock model created in this study had a mean absolute error of 9.5 years. Interestingly, this study found that species belonging to the *Ruminococcaceae*, *Bifidobacteriaceae*, *Lachnospiraceae*, and *Clostridiaceae* families were shown to have the greatest impact on the aging predictions in this model, and this is in agreement with the previously mentioned gut microbiome aging model created from 16S rRNA sequencing [18,23]. Additionally, this study found that there were several metabolic pathways that were negatively associated with aging, and these include vitamin B12 biosynthesis genes, amino acid metabolism genes, and SCFA production genes. These functional changes had the most influence on the aging clock predictions and have been validated by other studies to be age-related changes of interest [12,36].

#### 3.2.4. Meta-Metabolomic and Proteomic Clocks

There is growing interest in utilizing metabolomic profiling to correlate metabolic changes with the biological functions of host microbiomes, such as the gut microbiome. There have been some studies highlighting age-related metabolic changes, and some predictions have been created based on these. For example, a 2019 study found that metabolites most associated with their model of biological age included amino acids, fatty acids, acylcarnitine, nucleotide, and sphingolipid metabolites [37]. Other microbiome-derived metabolites found to be related to age include uremic toxins, indoles, and phenylacetylglutamine [21]. As previously mentioned, amino acid metabolism and SCFA production are intricately related to age as well, and these metabolites may also be quantified and correlated using meta-metabolomics [12,36]. Furthermore, proteomic analyses have emerged, and aging predictions based on microbial metabolomes and proteomes may be another future direction for microbiome-based aging clocks.

#### 3.2.5. Gut Microbiome Modulation of Other Aging Clocks

Examples of epigenetic aging clocks include the Hunnam clock, Horvath clock, PhenoAge clock, GrimAge clock, and DunedinPACE clock. The Hunnam clock was the earliest epigenetic aging clock and was curated based on CpG sites utilizing elastic net regression [4]. The Horvath clock estimated the DNA methylation of various tissue and cell types using a wider range of CpG islands [5]. Consequently, the PhenoAge clock, GrimAge clock, and DunedinPACE clock were created by expanding the populations and incorporating clinical biomarkers and lifestyle factors to refine the estimation of biological age [38,39,40]. The latter clocks are also unique in that they link phenotypic age and thus better reflect physiological conditions. Furthermore, aging clocks based on telomere length, exosomes, inflammation, and other blood biomarkers are emerging as well.

Gut microbiota may modulate epigenetic aging clock predictions directly and indirectly via the regulation of epigenetic changes. For example, one 2020 study found that exposure to microbiota in the setting of induced acute inflammation resulted in DNA hypomethylation and the upregulation of anti-microbial and anti-inflammatory genes [41]. In ulcerative colitis patients, high *Fusobacterium* levels were associated with increased DNA methylation in colorectal cancer-related genes [42]. Animal studies have shown that early shifts in microbial colonization are associated with altered DNA methylation and gene expression [43,44].

#### 3.2.6. Gut Microbiome-Derived Secondary Metabolites

Microbiota-derived metabolites may also serve as epigenetic substrates or regulate enzymes involved in epigenetic changes (Figure 1). For example, the metabolism of folate by *Bifidobacterium* and *Lactobacillus* species generates S-adenosylmethionine (SAM), the primary substrate for DNA and histone methylation [45]. Commensal bacteria may also metabolize polyphenols or methionine into SAM [46]. The production of SCFAs, such as butyric acid, acetic acid, and propionic acid, may regulate histone acetylation and non-coding RNAs [9,10]. It is thought that SCFAs and polyphenol metabolites may act as histone deacetylase (HDAC) inhibitors, thereby promoting histone acetylation and influencing gene expression [47]. SCFAs may also provide acyl-CoA precursors, and this may increase acetyl-CoA concentrations, leading to enhanced histone acetylation as well [48]. Moreover, the influence of the gut microbiome on other systems, such as the brain and skin, may also contribute to regulating other biological aging clocks, and further research on this topic is warranted.

### 3.3. Skin Microbiome Aging Clocks

To the best of our knowledge, there is only one skin microbiome aging clock studied thus far.

This 2020 study utilized the 16S rRNA sequencing data from a total of 1975 skin samples of healthy subjects aged 18 to 90 years to predict aging [19]. The study utilized random forests to regress the relative abundance of microbiota against the subjects’ chronological ages and found that the skin microbiota was correlated with chronological age, with a mean absolute error of 11.5 years. Among the taxa that contributed the most to the age prediction model included *Mycoplasma*, *Enterobacteriaceae*, and *Pasteurellaceae*, which were all negatively correlated with age [19]. Although some studies have shown that gut *Enterobacteriaceae* and oral *Pasteurellaceae* may be involved in the aging process [49,50,51], more research is necessary to determine the role of these microbes on the skin and how they may accelerate aging. This study also examined the oral and gut microbiome and found that the skin microbiome was more accurate in predicting chronological age, followed by the oral and gut microbiome, respectively [19]. However, these findings are limited by the utilization of 16S rRNA sequencing, and it may be interesting to investigate whether utilizing metagenomic sequencing and validating the results with functional data, metabolomics, or transcriptomics may produce secondary findings. Future research may also build on these results to create testing modalities that can estimate accelerated or delayed aging, thereby expanding the ability to modify and monitor the aging process.

#### 3.3.1. Skin Microbiome Modulation of Other Aging Clocks

To the best of our knowledge, there are no studies evaluating how the skin microbiome may influence epigenetic changes that modulate epigenetic aging clock predictions. However, the skin microbiome is known to be intricately involved in immune inflammatory responses, and this may be one way in which the skin microbiome may modulate aging clocks based on inflammatory markers. For example, the iAge clock is an inflammatory aging clock that is heavily influenced by markers such as CXCL9, EOTAXIN, IL-1β, IL-5, IFN-α, and IFN-γ, among others [52]. Skin microbiota mitigate atopic dermatitis severity, which is associated with pro-inflammatory cytokines, such as IL-5, and influence inflammatory dermatoses, such as rosacea, via IFN expression [53]. Taken together, the skin microbiome may be involved in the regulation of these aging clock contributors, and more research is needed.

#### 3.3.2. Skin Microbiome-Derived Secondary Metabolites

Secondary metabolites produced by cutaneous microbiota include short-chain fatty acids (SCFAs), tryptophan metabolites, amine derivatives, and antibiotics. They have been shown to have systemic effects on the immune system and are relevant to dermatological conditions such as atopic dermatitis and psoriasis [54,55]. Bacteria can synthesize and release compounds, including histamine, glutamate, and γ-aminobutyric acid or peptides [56], which interact with host cells to mediate skin and immune cell function. SCFAs produced by *Cutibacterium acnes* and *Staphylococcus epidermidis* are also found in the skin microbiome’s secondary metabolites [57], and these maintain an acid skin pH where symbionts flourish. Also, the postbiotic materials of SCFAs may act as an immune regulator against pathogens in microbial communities. To the best of our knowledge, there are no studies that elaborate on how these metabolites may interact with aging clocks. However, skin microbiome-derived secondary metabolites may influence aging clocks, and further investigation into this subject is warranted.

### 3.4. Technological Advances and Measurement Techniques

Advancements in the characterization of human microbiomes have evolved rapidly in recent years. The traditional standardized sequencing method for taxonomic characterization is 16S rRNA sequencing. However, this method is limited by its analysis of the 16S rRNA gene, which is only found in bacteria [58]. Next-generation sequencing techniques, including whole genome sequencing, have made it possible to classify the microbiome at deeper levels and expand taxonomic characterization to include fungi, viruses, phages, and more. Additionally, the potential to validate microbiome findings with the metabolome or meta transcriptome may further refine the current knowledge of the microbiome and its relationship with biological systems. Aging clocks and biological age prediction models are continuously and rapidly evolving. This is, in part, due to the development of biomedical research databases with very large sample sizes that have led to the expansion of biological age estimation models. Advancements in deep learning models have also accelerated the creation of these predictions.

### 3.5. Challenges and Limitations

Aging clocks have been shown to have the potential for the diagnosis of disease and prediction of biological aging in an individual; however, there are challenges and limitations to their use. One such challenge is the delineation of the chronological and biological components of the DNA methylation clocks. These clocks are currently better at estimating chronological age than other methods, such as transcriptomic, proteomic data, or telomere length [59]; however, there is still some variability in age estimation. The DNA methylation clocks are built with a supervised machine learning method trained against chronological age to identify an informative predictive set of CpGs [60]. The difference from chronological age is used as a marker for the biological age of an individual. The age-related phenotype may be due to disease, mortality, clinical measures of frailty, or cellular phenotypes, including the mitotic age of a tissue [61]. By increasing the training sample size, improved measurement of chronological age is expected [62]. During a study on aging in Hutchinson Gilford Progeria Syndrome (HGPS), it was found that epigenetic age changes in fibroblasts were detected with the Hannum skin and blood clock, whereas the pan-tissue clock by Horvath did not detect the age changes in this case. One of the challenges is to choose the correct type of testing for the type of study or research [63].

Another major challenge is to determine the individual contributing factors, how they interact, and their relative contributions to aging, with the final goal of identifying potential biochemical targets to address aging while having minimal side effects [64]. A further caution in the utilization of aging clocks is that much of our understanding of the biology of aging comes from studies on model organisms, such as mouse or baboon research data. Additional research involving human subjects is currently in progress to acquire a detailed understanding of the impact of the aging clock and potential interventions that can slow or reverse the aging process.

#### 3.5.1. Interindividual Variability

Despite high correlations, DNAmAge estimates can deviate substantially from chronological age at the individual level. Intrinsic age acceleration (IEAA), as derived from the Horvath measure of DNAmAge based on 353 CpGs [5], is independent of chronological age and variation in blood cell composition. On an individual level, epigenetic age acceleration (EEAA) is lowered with the body mass index (BMI) and responds to the protective effects of diet, exercise, and education, as well as the risk of obesity and dyslipidemia [65]. Also, studies have shown that interventions that significantly impact DNAmAge are dependent on population and cultural, gender, and individual genetic-specific factors [66].

#### 3.5.2. Standardization and Validation

Standardization and validation are important considerations in this emerging field of study. A challenge so far is the lack of human longitudinal studies. These studies are necessary to assess and validate epigenetic clock variation and divergence in an individual life span. Longitudinal studies can also better assess the predictive power of the various aging clocks [67]. Another issue is that all these genetic and epigenetic data and analyses are strongly biased toward populations of European ancestry, and other populations are under-represented, so the extent of genetic influence is currently underestimated. Therefore, further large-scale, diverse longitudinal studies and detailed analyses across multiple populations are imperative [68].

#### 3.5.3. Ethical Considerations

Ethical consideration must be given to the research process itself in both animal and human research studies and its resulting use in medicine. In the case of the medical application of aging clocks, Mackey describes six potential ethical considerations: (1) inequity whereby the wealthy will be better able to access treatments, (2) denying the immutability of aging, (3) dominating nature and commodifying humans, (4) overpopulation, (5) ennui, and (6) ageism [69]. These areas of concern must be weighed against the potential benefits of a longer and healthier life while ensuring that these benefits are available to all. Another ethical concern is the potential misuse of epigenetic age estimators by private and public organizations. Many private companies now sell epigenetic tests directly to consumers online [70]. Another ethical consideration is the use of informed consent for the further use of these data sets. One potential misuse is in insurance applications whereby individuals may be declined insurance or subjected to increased rates due to adverse risk factors. There is also the possibility of genetic profiling in forensic or immigration applications [70].

### 3.6. Clinical Implications and Future Directions

Continued research into aging clocks may allow targeted and personalized or population-based therapeutic interventions. The future of this research appears to be in combining all data through metagenomics and deep learning into an aging clock model and prescribing testing interventions that can slow or reverse the aging process. However, the mechanisms of aging are a complex interconnected web of genetic and epigenetic factors. Further study of aging clocks will facilitate future interventions for improving human health and longevity. These might include epigenetic drugs; stem cell-based strategies; the clearance of senescent cells; DR, IIS, and mTOR inhibition; and AMPK and sirtuins activation [64]. The future may lie in new technologies and deep learning applications. A few sequencing-based studies of chronological aging clocks have investigated regions beyond the CpGs profiled using array-based techniques. It may not be cost-effective to use deep sequencing-based studies, especially with whole-genome base-resolution techniques where Horvath’s pan-tissue clock exploits the 27k array and is highly accurate in predicting chronological age [62]. However, research into epigenetic factors that slow the aging clock will continue to expand our understanding and lead to new interventions and treatments. For example, studies that focused on reversing the DNA methylation profile brought to light a class of drugs called epidrugs, which lead to changes in gene expression. Some DNA methyltransferase inhibitors (DNMTis) have been approved by the FDA and are in use in oncological diseases [71].

Another potential is the development of novel aging clocks. In 2020, a skin-specific biological clock was developed using data from 508 human skin samples [72]. Another potential clock is the “facial aging clock”, as proposed by Wang et al. This clock relies on deep learning-based facial image technologies and is less invasive and expensive than the DNA methylation clock. They also purport to provide an objective identification of disease risk and progression and an evaluation of ongoing therapies [73]. The future of aging clocks will depend on the integration of all available data into a single reliable model to allow for a better understanding of the role of the microbiome in disease, with the goal of discovering novel and effective therapies.

## 4. Conclusions

Human gut and skin microbiomes may be utilized based on taxonomy, biodiversity, and functionality to create aging clock predictions. Furthermore, gut and skin microbiota may influence clock genes that regulate other biological and epigenetic aging clocks through the metabolism and production of secondary metabolites, among other mechanisms. Future directions include integrating metabolomic, proteomic, and transcriptomic data into microbiome-based aging clocks and the expansion of sequencing techniques and deep learning models to refine these predictions. Overall, the ability to monitor and modify biological age remains an ongoing challenge to combat disease and age-related changes in physiology and functionality.

## Figures and Tables

**Table 1:** Summary of microbiome-based aging clocks.

Author (Year)	Type of Aging Clock	Gut or Skin Microbiome	Key Findings
Galkin et al. (2020) [18]	Taxonomic	Gut	*Bifidobacterium* spp., *Akkermansia muciniphila*, and *Bacteroides* spp. or pathogenic bacteria, such as *Escheria coli* and *Campylobacter jejuni*, were most predictive.
Huang et al. (2020) [19]	Taxonomic	Gut	*Bifidobacterium* and *Blautia* or the families *Lachnospiraceae*, *Ruminococcaceae*, and *Clostridiaceae* had the highest feature importance scores.
Sala et al. (2020) [20]	Biodiversity	Gut	There was a decrease in microbial diversity with unhealthy phenotypes at older ages.
Wilmanski et al. (2021) [21]	Biodiversity	Gut	The β-diversity of gut microbiomes becomes more unique with age and is related to metabolomic changes.
Chen et al. (2022) [22]	Functional	Gut	Acetyl-CoA biosynthesis and nicotinate degradation were the most predictive. *Finegoldia magna*, *Bifidobacterium dentium*, and *Clostridium clostridioforme* increased, while *Prevotella copri* and *Burkholderialse* bacterium decreased with age.
Gopu et al. (2024) [23]	Functional	Gut	*Ruminococcaceae*, *Bifidobacteriaceae*, *Lachnospiraceae*, and *Clostridiaceae* families were the most predictive.
Huang et al. (2020) [19]	Taxonomic	Skin	*Mycoplasma*, *Enterobacteriaceae*, and *Pasteurellaceae* were negatively correlated with age.

**Table 2:** Microbiome-based aging clocks and predictability against chronological age.

Author (Year)	Type of Aging Clock	Gut or Skin Microbiome	Type of Sequencing	R^2^	Mean Absolute Error (Years)
Galkin et al. (2020) [18]	Taxonomic	Gut	Metagenomic	0.20	10.6
Huang et al. (2020) [19]	Taxonomic	Gut	16S rRNA	0.17	11.5
Sala et al. (2020) [20]	Biodiversity	Gut	16S rRNA	*	*
Wilmanski et al. (2021) [21]	Biodiversity	Gut	16S rRNA	*	*
Chen et al. (2022) [22]	Functional	Gut	Metagenomic	0.6	8.3
Gopu et al. (2024) [23]	Functional	Gut	Metagenomic	0.42	9.5
Huang et al. (2020) [19]	Taxonomic	Skin	16S rRNA	0.74	3.8

All studies listed were performed utilizing human samples. * Study was included to demonstrate that biodiversity-based aging clocks are one subset of microbiome-based aging clocks. However, these studies did not perform any error descriptions or correlation analyses.

## Data Availability

No new data were generated for this review, but studies were reviewed from publicly available databases.
